# Isolation and Structural Determination of the First 8-*epi-*type Tetrodotoxin Analogs from the Newt, *Cynops ensicauda popei, *and Comparison of Tetrodotoxin Analogs Profiles of This Newt and the Puffer Fish, *Fugu poecilonotus *

**DOI:** 10.3390/md10030655

**Published:** 2012-03-22

**Authors:** Yuta Kudo, Takeshi Yasumoto, Keiichi Konoki, Yuko Cho, Mari Yotsu-Yamashita

**Affiliations:** 1 Graduate School of Agricultural Science, Tohoku University, 1-1 Tsutsumidori-Amamiyamachi, Aoba-ku, Sendai 981-8555, Japan; Email: b1am1315@s.tohoku.ac.jp (Y.K.); konoki@biochem.tohoku.ac.jp (K.K.); choyuko@biochem.tohoku.ac.jp (Y.C.); 2 Tama Laboratory, Japan Food Research Laboratories, 6-11-10 Nagayama, Tama-shi, Tokyo 206-0025, Japan; Email: yasumotot@jfrl.or.jp

**Keywords:** tetrodotoxin, 8-*epi*-5,6,11-trideoxytetrodotoxin, puffer fish, newt, LC/MS

## Abstract

Identification of new tetrodotoxin (TTX) analogs from TTX-possessing animals might provide insight into its biosynthesis and metabolism. In this study, four new analogs, 8-*epi*-5,6,11-trideoxyTTX, 4,9-anhydro-8-*epi*-5,6,11-trideoxyTTX, 1-hydroxy-8-*epi*-5,6,11-trideoxyTTX, and 1-hydroxy-4,4a-anhydro-8-*epi*-5,6,11-trideoxyTTX, were isolated from the newt, *Cynops ensicauda popei*, and their structures were determined using spectroscopic methods. These are the first 8-*epi*-type analogs of TTX that have been found in a natural source. Furthermore, we examined the composition of the TTX analogs in this newt and in the ovary of the puffer fish, *Fugu poecilonotus*, using LC/MS. The results indicate that TTX and 11-deoxyTTX were present in both sources. However, 6-*epi*TTX and 8-*epi*-type analogs were detected only in the newt, while 5,6,11-trideoxyTTX was a specific and major analog in the puffer fish. Such considerable differences among analog compositions might reflect differences in the biosynthesis or metabolism of TTX between these animals.

## 1. Introduction

**Figure 1:**
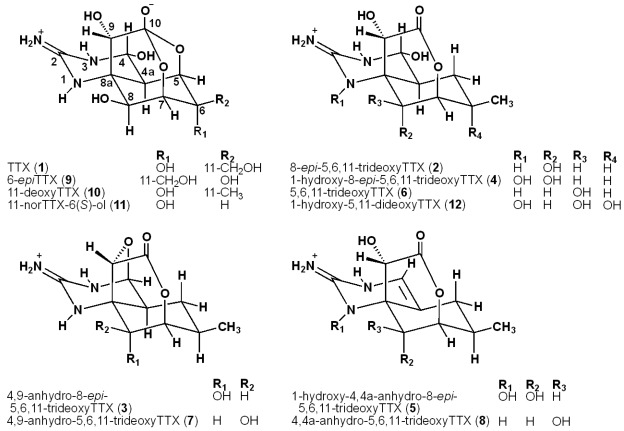
The structures of Tetrodotoxin (TTX) (**1**) and its analogs found in newts (**2**–**5**, **9**, **10**, **12**) and in puffer fishes (**6**–**8**, **10**, **11**).

Tetrodotoxin (TTX, **1**, Figure 1), a well-known potent neurotoxin, was first isolated from puffer fish [1,2,3] and was also found in newts (*Taricha*, *Cynops*, *Notophthalmus*, *Triturus*, and *Paramesotriton*), salamanders (*Ambystoma*) [4,5,6,7], and frogs (*Atelopus* and some other species) [8,9,10]. TTX is believed to function as an antipredator mode of defense in newts, and their toxicity has been used to study the evolution and chemical ecology of TTX [11].

Several TTX analogs have been found in newts and puffer fish. We first isolated 6-*epi*TTX (**9**) and 11-deoxyTTX (**10**) from the newt, *Cynops ensicauda* (*Cynops ensicauda popei*, at present) [12], and reported that 6-*epi*TTX is a major TTX analog in some species of newts [6]. On the other hand, 1-hydroxy-5,11-dideoxyTTX (**12**) was isolated from the newt *Taricha granulosa* by Kotaki and Shimizu as the first 1-(*N*)-hydroxy 10,7-lactone type analog [13]. From puffer fish, we isolated 5-deoxyTTX, 6,11-dideoxyTTX, 5,6,11-trideoxyTTX (**6**), 11-norTTX-6(*S*)-ol (**11**), and 4-*S*-cysteinylTTX [14,15,16,17,18], and we demonstrated that 5,6,11-trideoxyTTX (**6**) is a major analog in puffer fish using hydrophilic interaction liquid chromatography-electrospray ionization/mass spectrometry (HILIC-ESI/MS) to analyze TTX analogs [19,20,21,22]. However, we have not examined whether these analogs are specifically present in newts or in puffer fish or if they are commonly present in both of them. 

Puffer fish is assumed to accumulate TTX that is produced by bacteria via the food chain or by symbiotic bacteria because many TTX-producing bacteria have been reported from marine sources [23,24,25]. However, for newts, the origin of TTX is still controversial because TTX-producing bacteria have not yet been identified in terrestrial areas [26]. On the other hand, according to Shimizu *et al.* [27], radiolabeled compounds administrated to *T. granulosa* were not incorporated into TTX. Thus, as an alternative approach, we have continued screening TTX analogs in newt and puffer fish because we believe that the chemical structures of TTX analogs might reflect the process of biosynthesis or metabolism of TTX. 

We report here the isolation and structural determination of four new TTX analogs, 8-*epi*-5,6,11-trideoxyTTX (**2**), 4,9-anhydro-8-*epi*-5,6,11-trideoxyTTX (**3**), 1-hydroxy-8-*epi*-5,6,11-trideoxyTTX (**4**), and 1-hydroxy-4,4a-anhydro-8-*epi*-5,6,11-trideoxyTTX (**5**) from the newt *C. ensicauda popei*. Furthermore, we quantified several TTX analogs from both the newt and the puffer fish, *Fugu poecilonotus*, using LC/MS to compare the TTX profiles of newt and puffer fish. We discuss the significance of these results with respect to the biosynthetic or metabolic pathways of TTX.

## 2. Results and Discussion

### 2.1. Purification of New TTX Analogs and Their Molecular Formulas

The extract of the whole body of* Cynops ensicauda popei* revealed four unknown peaks on the LC/MS/MS (MRM) mass chromatograms. These peaks, detected at *m/z* 288–162, 272–162, 270–162, and 254–162, corresponded to **2**, **3**, **4**, and **5**, respectively. The compounds were isolated by column chromatography according to the procedure outlined in the experimental section. The approximate yields of **2**, **3**, **4,** and **5** were 0.35, 0.25, 0.06 and 1.2 mg, respectively, from 170 g of *C. e. popei*. Their molecular formulas were determined by high resolution-fast atom bombardment mass spectrometry (HR-FABMS), the results of which suggest that these compounds are TTX analogs. Positive, *m/z* [M + H]^+^, **2**: found 272.1248, calcd for C_11_H_18_O_5_N_3_ 272.1241; **3**: found 254.1137, calcd for C_11_H_16_O_4_N_3_ 254.1135; **4:** found 288.1200, calcd for C_11_H_18_O_6_N_3_ 288.1190; **5**: found 270.1094, calcd for C_11_H_16_O_5_N_3_ 270.1085.

### 2.2. The Structures of 8-*epi*-5,6,11-trideoxyTTX (2) and 4,9-Anhydro-8-*epi*-5,6,11-trideoxyTTX (3)

**2 **(C_11_H_17_O_5_N_3_) has the same molecular formula as 5,6,11-trideoxyTTX (**6**). The structural determinations of **2** and **3** were achieved mainly through NMR measurements. Assignments of all ^1^H and ^13^C signals of **2** were derived from ^1^H–^1^H COSY, TOCSY, HSQC, and HMBC, except C2 (Table 1). ^1^H–^1^H COSY of **2** showed couplings between H4/H4a, H4a/H5ax, H4a/H5eq, H5ax/H5eq, H5ax/H6, H5eq/H6, H6/Me11, and H7/H8, analogous with 5,6,11-trideoxyTTX (**6**) [16]. The HMBC spectrum of **2** clarified the connectivities around quaternary carbons at C8a and C10 by giving cross-peaks due to C5/Me11, C6/Me11, C6/H4a, C6/H8, C4a/H9, C8a/H7, C8a/H9, C7/Me11, and C10/H9. Although the HMBC correlation with C2/H4 was not found, most likely because of the small sample amount, the presence of a guanidinium group in **2** was strongly suggested by the molecular formula and chromatographic properties. These data suggest that **2** is an epimer of 5,6,11-trideoxyTTX.

**Table 1:** ^13^C and ^1^H NMR data of 8-*epi*-5,6,11-trideoxyTTX (**2**) and 5,6,11-trideoxyTTX (**6**) [16] in CD_3_COOD-D_2_O (4:96, v/v).

	8-*epi*-5,6,11-trideoxyTTX (2)		5,6,11-trideoxyTTX (6)		Δδ (2–6)	
Position	δ_C_	δ_H_ (*J* in Hz)	δ_C_	δ_H_ (*J* in Hz)	Δδ_C_	Δδ_H_
2	ND		155.7			
4	77.6	5.17, d (8.8)	77.3	5.17, d (10.0)	0.3	0.00
4a	40.6	2.28, m	46.2	1.99, ddd (13.3, 10.0, 3.9)	−5.6	0.29
5eq	27.5	2.05, m	27.8	2.07, dt (13.3, 4.0)	−0.3	−0.02
5ax		0.87, q (13.3)		0.92, q (13.3)		−0.05
6	32.1	2.30, m	36.9	2.13, m	−4.8	0.17
7	83.9	4.55, br s	87.2	4.61, br t	−3.3	−0.06
8	67.9	4.33, d (4.1)	74.5	4.10, d (2.3)	−6.6	0.23
8a	61.4		61.2		0.2	
9	75.0	4.36, s	72.4	4.63, s	2.6	−0.27
10	176.6		177.4		−0.8	
11	17.8	1.06, d (7.0)	18.3	1.08, d (6.7)	−0.5	−0.02

ND denotes not determined.

**Figure 2:**
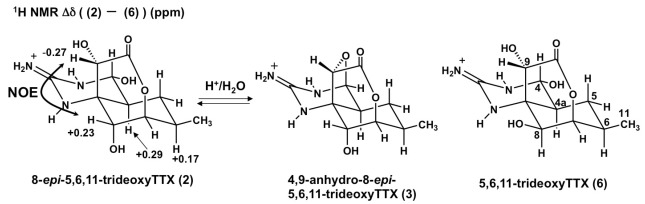
The structures of the new TTX analogs (**2**, **3**) from the newt and their structurally similar analog (**6**) from puffer fish. The structure of **2** is shown with the difference of ^1^H NMR chemical shifts between **2** and **6**, and key NOEs.

Comparing the ^13^C and ^1^H NMR signals of 5,6,11-trideoxyTTX (**6**) to those of **2** (Table 1), large downfield shifts (ppm) of H4a (0.29), H6 (0.17), and H8 (0.23) as well as an upfield shift of H9 (−0.27) were observed, suggesting an equatorial substitution of H8 in **2 **(Figure 2). Large upfield shifts of C4a (−5.6), C6 (−4.8), and C8 (−6.6) and a downfield shift of C9 (2.6) supported the 8-*epi* assignment. NOE measurements by NOESY1D spectra confirmed the equatorial substitution of H8; irradiation at δ 4.33 (H8) enhanced the signal intensity of H9 (δ 4.36) in **2 **(Figure 2), while no enhancements were shown for H4a and H6 signals. In contrast, a positive NOE was observed on H6 when the signal at δ 4.10 (H8) was irradiated [16] for **6**. All these data support the structural assignment of **2**. It is the first identification of an 8-*epi*-type analog of TTX from a natural source. 

**3** was suggested to be 4,9-anhydro type of **2** based on its molecular formula (C_11_H_15_O_4_N_3_) and its assigned NMR signals from NMR data of ^1^H–^1^H COSY, TOCSY, and HSQC, except C2, C6, C8a, and C10 (Table 2). The singlet H4 signal and large downfield shifts of H4a (0.44) and H9 (0.68) compared with those of **2** were similarly observed, as they were in 4,9-anhydroTTX compared with TTX [28]. Comparing the NMR signals of 4,9-anhydro-5,6,11-trideoxyTTX (**7**) [29], downfield shifts of H6 (0.30–0.38) and H8 (0.17) and upfield shifts of C4a (−3.0) and C8 (−3.7) were observed, similar to those of **2**. Moreover, partial conversion of **2** to **3** was observed using LC/MS after incubation of **2** in 5% TFA/H_2_O (v/v) at 37 °C for 12 h, which strongly supports that **3** is the 4,9-anhydro type of **2**. The existence of a 4-*epi* analog of 8-*epi*-5,6,11-trideoxyTTX was also suggested by ^1^H–^1^H COSY, although their signals were not completely assigned due to a limited amount of compound.

**Table 2:** ^13^C and ^1^H NMR data of 4,9-anhydro-8-*epi*-5,6,11-trideoxyTTX (**3**) and 4,9-anhydro-5,6,11-trideoxyTTX (**7**) [29] in CD_3_COOD-D_2_O (4:96, v/v).

	4,9-Anhydro-8- *epi*-5,6,11-trideoxyTTX (3)		4,9-Anhydro-5,6,11-trideoxyTTX (7)		Δδ (3–7)	
Position	δ_C_	δ_H _(*J* in Hz)	δ_C_	δ_H _(*J* in Hz)	Δδ_C_	Δδ_H_
2	ND		156.0			
4	86.6	5.24, s	85.7	5.21, s	0.9	0.03
4a	40.7	2.72, dt (11.4, 6.5)	43.7	2.67, dd (11.2, 7.0)	−3.0	0.05
5eq	27.9	2.12, m	26.6	2.12–2.04, m	1.3	0.00–0.08
5ax		0.82, dt (15.9, 12.4)		0.82, q (11.2)		0.00
6	ND	2.42, m	33.8	2.12–2.04, m		0.30–0.38
7	84.0	4.60, br s	86.7	4.70, br s	−2.7	−0.10
8	64.3	4.60, br s	68.0	4.43, d (2.1)	−3.7	0.17
8a	ND		62.6			
9	85.6	5.04, s	83.5	4.98, s	2.1	0.06
10	ND		178.2			
11	18.7	1.03, d (6.8)	18.2	1.02, d (6.6)	0.5	0.01

ND denotes not determined.

### 2.3. The Structures of 1-Hydroxy-8-*epi*-5,6,11-trideoxyTTX (4) and 1-Hydroxy-4,4a-anhydro-8-*epi*-5,6,11-trideoxyTTX (5)

The ^13^C and ^1^H signals of **4** that were assigned based on ^1^H–^1^H COSY, TOCSY, HSQC and HMBC (Table 3) suggested that **4** is also a 5,6,11-trideoxy-type analog of TTX. Because the molecular formula of **4** (C_11_H_17_O_6_N_3_) has one more oxygen than that in 5,6,11-trideoxyTTX (**6**), we predicted an *N*-hydroxy guanidinium moiety in **4**, similar to the 1-hydroxy-5,11-dideoxyTTX found from *Taricha granulosa *[13]. Furthermore, in 5% TFA/H_2_O (v/v) at 37 °C, **4** gradually converted into **5**, which was suggested to be an anhydro analog of **4** by its molecular formula (C_11_H_15_O_5_N_3_). Because the yield of **5** (1.2 mg) was much higher than that of **4 **(0.06 mg), the structure of **5** was determined first. All ^13^C and ^1^H signals of **5** were assigned using the data obtained by ^1^H–^1^H COSY, TOCSY, HSQC, and HMBC (Table 4). The presence of the 4,4a-anhydro moiety was suggested by the chemical shifts of H4 (6.30), C4 (122.2), and C4a (110.3); by the appearance of the H4 signal as a singlet; and by the lack of a H4a signal. On the NMR spectra of the fraction of 4-*epi*-5,6,11-trideoxyTTX previously obtained from puffer fish [16], we also found similar signals assignable to 4,4a-anhydro-5,6,11-trideoxyTTX (**8**) as a minor component. Thus, the NMR signals of **8** were assigned and compared with those of **5** (Table 4). 

**Table 3:** ^13^C and ^1^H NMR data of 1-hydroxy-8-*epi*-5,6,11-trideoxyTTX (**4**) and 5,6,11-trideoxyTTX (**6**) in CD_3_COOD-D_2_O (4:96, v/v).

	1-Hydroxy-8- *epi*-5,6,11-trideoxyTTX (4)		Δδ (4–6)	
Position	δ_C_	δ_H _(*J* in Hz)	Δδ_C_	Δδ_H_
2	ND			
4	77.3	5.08, d (9.4)	0.0	−0.09
4a	40.3	2.54, dd (9.4, 2.3)	−5.9	0.55
5eq	28.2	2.05, m	0.4	−0.02
5ax		0.90, q (13.5)		−0.02
6	31.8	2.32, m	−5.1	0.19
7	84.2	4.67, d (4.4)	−3.0	0.06
8	65.3	4.53, d (4.1)	−9.2	0.43
8a	68.0		6.8	
9	69.0	4.79, s	−3.4	0.16
10	177.9		0.5	
11	17.5	1.06, d (7.0)	−0.8	−0.02

ND denotes not determined.

**Table 4:** ^13^C and ^1^H NMR data of 1-hydroxy-4,4a-anhydro-8-*epi*-5,6,11-trideoxyTTX (**5**) and 4,4a-anhydro-5,6,11-trideoxyTTX (**8**) in CD_3_COOD-D_2_O (4:96, v/v).

	1-Hydroxy-4,4a-Anhydro-8-*epi*-5,6,11-trideoxyTTX (5)		4,4a-Anhydro-5,6,11-trideoxyTTX (8)		Δδ (5–8)	
Position	δ_C_	δ_H _(*J* in Hz)	δ_C_	δ_H _(*J* in Hz)	Δδ_C_	Δδ_H_
2	154.6		152.4		2.2	
4	122.2	6.30, s	120.4	6.24, s	1.8	0.06
4a	110.3		110.1		0.2	
5eq	30.9	2.38, dd (15.8, 5.3)	29.9	2.37, d (14.1)	1.0	0.01
5ax		1.65, dd (15.0, 12.0)		1.65, t (13.6)		0.00
6	31.6	2.28, m	35.6	2.03, m	−4.0	0.25
7	84.1	4.67, d (4.1)	86.6	4.66, br s	−2.5	0.01
8	65.5	4.52, d (4.4)	73.3	4.27, br s	−7.8	0.25
8a	72.2		62.8		9.4	
9	68.8	4.80, s	71.2	4.48, s	−2.4	0.32
10	176.7		175.5		1.2	
11	17.9	1.04, d (6.8)	17.3	1.05, d (5.3)	0.6	−0.01

**Figure 3:**
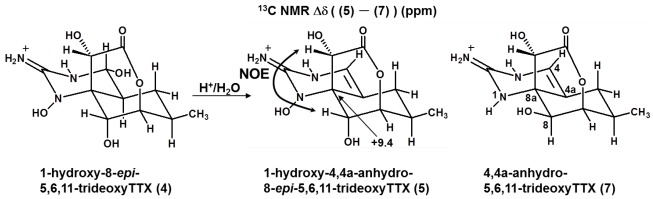
The structures of the new TTX analogs from the newt (**4**, **5**) and a structurally similar analog (**7**) from puffer fish. The structure of **5** is shown with the difference of ^13^C NMR chemical shifts between **5** and **7**, and key NOEs.

The downfield shifts of H6 (0.25), H8 (0.25), H9 (0.32), and C8a (9.4) and the upfield shifts of C6 (−4.0), C7 (−2.5), C8 (−7.8), and C9 (−2.4) of **5** were compared to those of **8**. In particular, the large downfield shift of C8a (9.4) indicated the presence of a hydroxyl group at the 1(*N*)-position of the guanidinium group in **5** because similar downfield shifts at the carbons neighboring the hydroxylated nitrogens were also observed in 1-hydroxy-5,11-dideoxyTTX (**12**) [13] and neosaxitoxin [30]. In 1-hydroxy-5,11-dideoxyTTX (**12**), the C8a signal (74.1) is 12.6 ppm downfield from the corresponding carbon in 5,11-dideoxyTTX (synthesized analog) [31], and the signal of C6 in neosaxitoxin, neighboring the *N*-hydroxy nitrogen, is 11.2 ppm downfield compared to saxitoxin [30]. In addition, downfield shifts of H6 (0.25) and H8 (0.25) as well as the upfield shifts of C6 (−4.0) and C8 (−7.8) were observed to be similar to the shifts in **2,** suggesting that **5** is also an 8-*epi*-type analog. In the NOESY1D spectra of **5**, positive NOEs were observed on H9 and H8, when the signals of H8 and H9 were irradiated, respectively, supporting the equatorial configuration of H8 and the same stereochemistry of C9 as that in TTX (Figure 3). All these data support the structural assignment of **5** as 1-hydroxy-4,4a-anhydro-8-*epi*-5,6,11-trideoxyTTX. In the ^1^H NMR spectrum of **4**, the two doublet protons at 2.54 and 5.08 ppm couple each other at 9.4 Hz and were assigned as H4/H4a protons (Table 3). These data and the fact that **4** was partially converted to **5** by incubation in 5% TFA/H_2_O (v/v) (Figure 3) strongly suggested that **4** is 1-hydroxy-8-*epi*-5,6,11-trideoxyTTX. 4-*epi* and 4,9-anhydro forms of **4** were not clearly detected by LC/MS or by NMR, most likely due to their low concentrations.

### 2.4. Comparison of TTX Analogs Profiles of the Newt, *C. e. popei* and the Puffer Fish, *Fugu poecilonotus*

**Figure 4:**
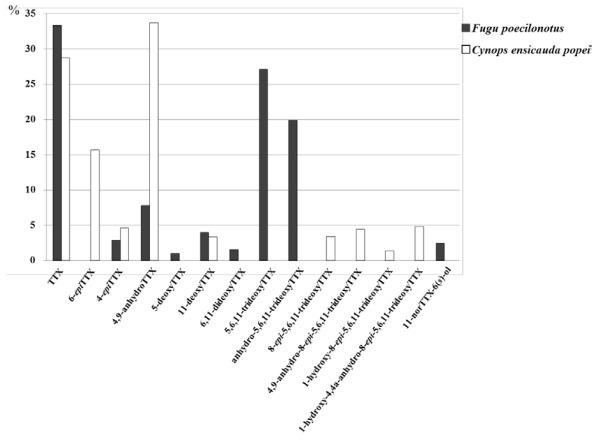
The profiles of TTX analogs in the whole body of *Cynops ensicauda popei* and the ovary of *Fugu poecilonotus*. Total mol of TTX analogs is counted as 100%.

The ratios of each TTX analog to total TTXs in the pooled whole body of the newt, *C. e. popei*, and in the pooled ovary of the puffer fish, *Fugu poecilonotus*, was determined by qualitative/quantitative analysis using LC/MS (Figure 4). In both animals, TTX was the major component among the analogs, composing more than 33.4% in the puffer fish and 28.7% in the newt. Additionally, 11-deoxyTTX was detected in both animals. However, the presence of the other TTX analogs greatly differed between puffer fish and newt. The 8-*epi*-5,6,11-trideoxyTTX-type analogs **2**, **3**, **4**, and **5 **were not detected in puffer fish. Instead, 5,6,11-trideoxyTTX (**6**) was a major analog (27.1%) in puffer fish. Conversely, **6** was not detected in newt. Additionally, 11-norTTX-6(*S*)-ol, a minor analog of TTX in puffer fish (2.5%), was not detected in newt. Moreover, 6-*epi*TTX, a major analog in newt (15.7%), was not detected in puffer fish. The characteristic difference in the TTXs profiles of newt and puffer fish was confirmed by our results. The toxin contents per gram of ovary of the puffer fish and whole body of the newt were listed in Table 5. 

**Table 5:** The contents of TTX analogs per gram of the whole body of *Cynops ensicauda popei* and the ovary of *Fugu poecilonotus.*

Toxin	LC/MS Results (μg/g)	
	*F. poecilonotos*	*C. e. popei*
TTX	134.0	49.6
6-*epi*TTX	<0.3	27.0
4-*epi*TTX	11.5	8.0
4,9-anhydroTTX	31.2	58.1
5-deoxyTTX	4.2	<0.3
11-deoxyTTX	15.9	5.7
6,11-dideoxyTTX	6.3	<0.3
5,6,11-trideoxyTTX	108.8	ND *
anhydro-5,6,11-trideoxyTTX	79.9	ND *
8-*epi*-5,6,11-trideoxyTTX	ND *	5.8
4,9-anhydro-8-*epi*-5,6,11-trideoxyTTX	ND *	7.7
1-hydroxy-8-*epi*-trideoxyTTX	<0.3	2.4
1-hydroxy-4,4a-anhydro-8-*epi*-5,6,11-trideoxyTTX	<0.3	8.3
11-norTTX-6(*S*)-ol	9.9	<0.3

ND denotes not detected; * 5,6,11-TrideoxyTTX and 8-*epi*-5,6,11-trideoxyTTX were not clearly distinguished with each other by HILIC/MS. ^1^H NMR was used to identify these analogs in the corresponding fractions.

### 2.5. Discussion

TTX and its analogs are distributed in a wide range of marine and terrestrial animals, such as puffer fish, blue-ringed octopus, crabs, flatworms, snails, frogs, and newts. We have isolated various TTX analogs from these animals and attempted to obtain clues to the biosynthetic and/or metabolic pathways of TTX from the chemical structures of these analogs. In the present study, we isolated and determined the structures of the new TTX analogs 8-*epi*-5,6,11-trideoxyTTX (**2**), 4,9-anhydro-8-*epi*-5,6,11-trideoxyTTX (**3**), 1-hydroxy-8-*epi*-5,6,11-trideoxyTTX (**4**), and 1-hydroxy-4,4a-anhydro-8-*epi*-5,6,11-trideoxyTTX (**5**) derived from the newt *Cynops ensicauda popei*. Our preliminary examination using LC/MS suggested that **2 **is also present in species of newt other than *C. e. popei*. 

8-*epi*-5,6,11-trideoxyTTX (**2**) seems to be specific to newts, while 5,6,11-trideoxyTTX (**6**) is a specific and major analog in puffer fish (and in some other marine animals). 1-Hydroxy-type analogs of TTX also seem to be specific to newts because another 1-hydroxy analog, 1-hydroxy-5,11-dideoxyTTX (**12**), was isolated from the newt *Taricha granulosa *by Kotaki and Shimizu [13]. As previously suggested [6,7], 6-*epi*TTX is a major and specific analog in newts and is not detected in puffer fish. The present study confirmed different TTX analog profiles between newts and puffer fish 

We found a series of deoxy analogs such as 5,6,11-trideoxyTTX (**6**) [16], 6,11-dideoxyTTX [15], 5-deoxyTTX [14], and 11-deoxyTTX (**10**) [12] in puffer fish. We hypothesized that TTX might be derived from 5,6,11-trideoxyTTX via step by step oxidation in TTX-producing marine bacteria that accumulate in puffer fish [22]. However, the highly toxic newt, *C. e. popei*, does not contain 5,6,11-trideoxyTTX and, surprisingly, contains only its 8-epimer. We also searched 8-*epi*TTX in TTX fractions semi-purified from *C. e. popei*, using NMR (^1^H–^1^H COSY). Although a slight cross peak at 5.54/2.53 ppm, which was suspected to correspond to H4/H4a of 8-*epi*TTX (*cf*. H4/H4a of TTX is shown at 5.50/2.35 ppm), was observed, only trace amounts of 8-*epi*TTX should be in the newt, if at all. Accordingly, we have no experimental evidence that 1(*N)*-hydroxyTTX is present in the newt. 

The origin of TTX in newts, that is, whether its origin is “external or internal,” is still unresolved [11]. In the present study, additional newt-specific TTX analogs were identified and showed different toxin profiles in newts versus puffer fish. However, these data do not refute the possibility of an external origin of TTX in newts. For example, the analogs of saxitoxin, another well-known and widely distributed guanidinium toxin, are produced both by marine dinoflagellates [32] and by terrestrial (freshwater) cyanobacteria [33]. However, freshwater cyanobacteria produce specific saxitoxin analogs (acetylated types) [34] that have not been reported to be produced by marine dinoflagellates and shellfish. Additional studies to investigate the origin of TTX in newts need to be carried out in the future. 

## 3. Experimental Section

### 3.1. Purification of 8-*epi*-5,6,11-trideoxyTTX (2), 4,9-Anhydro-8-*epi*-5,6,11-trideoxyTTX (3), 1-Hydroxy-8-*epi*-5,6,11-trideoxyTTX (4), and 1-Hydroxy-4,4a-Anhydro-8-*epi*-5,6,11-trideoxyTTX (5)

The newts of the species *Cynops ensicauda popei* (170 g) were collected in Okinawa, Japan, frozen, and stored below −15 °C until use. The whole body was homogenized, extracted with 0.2 M acetic acid (340 mL) by heating for 10 min in boiling water, and centrifuged for 15 min at 15,000 rpm at 4 °C. The supernatant was diluted with water (1 L), and defatted with hexane (1 L). The extract, adjusted to pH 6.0 by 1 M NaOH, was loaded on an activated charcoal column (30 i.d. × 215 mm) equilibrated with water. After the column was washed with water, TTXs were eluted with acetic acid/EtOH/water (2:50:49, v/v). The eluate was concentrated by evaporation of the solvent, and then successively chromatographed on the weak cation exchange columns of Bio-Rex 70 (10 i.d. × 160 mm, 200–400 mesh, Bio Rad, Hercules, CA, USA) and Hitachi gel 3011C (7.0 i.d. × 300 mm), all equilibrated with water before use. Elution of TTX analogs from the columns was monitored by LC/MS. TTX analogs that were retained on the columns with water were eluted with 0.2 M acetic acid and 0.5 M acetic acid from Bio-Rex 70 and Hitachi gel 3011C, respectively. For further purification, new TTXs were purified on a TSK gel G1000PW (8.0 i.d. × 600 mm, Tosoh, Tokyo, Japan) with 0.05 M AcOH. Finally, pure 1-hydroxy-8-*epi*-5,6,11-trideoxyTTX (**4**, approximately 60 µg, by ^1^H NMR), 1-hydroxy-4,4a-anhydro-8-*epi*-5,6,11-trideoxyTTX (**5**, approximately 1.2 mg), 8-*epi*-5,6,11-trideoxyTTX (**2**, approximately 350 µg), and 4,9-anhydro-8-*epi*-5,6,11-trideoxyTTX (**3**, approximately 250 µg) were obtained. These compounds were analyzed by high resolution (HR)-fast atom bombardment (FAB)-MS and NMR analysis.

### 3.2. LC/MS and LC/MS/MS

LC/MS and LC/MS/MS were performed based on HILIC as we reported previously [20,21,22]. LC/MS and LC/MS/MS experiments were recorded on an API2000 mass spectrometer (AB SCIEX, Foster City, CA, USA) equipped with an ESI source. Nine ions at *m/z* 254, 270, 272, 286, 288, 290, 302, 304, and 320, corresponding to the [M + H]^+^ ions of TTX analogs, were detected in single ion monitoring (SIM) mode. The *m/z* 320–162 corresponding to TTX, 4-*epi*TTX, and 6-*epi*TTX; *m/z* 304–162 corresponding to 5-deoxyTTX and 11-deoxyTTX; *m/z* 302–162 corresponding to 4,9-anhydroTTX; *m/z* 288–162 corresponding to 1-hydroxy-8-*epi*-5,6,11-trideoxyTTX; *m/z* 288–224 corresponding to 6,11-dideoxyTTX; *m/z* 272–162 corresponding to 5,6,11-trideoxyTTX and 8-*epi*-5,6,11-trideoxyTTX; *m/z* 270–162 corresponding to 1-hydroxy-4,4a-anhydro-8-*epi*-5,6,11-trideoxyTTX; and 254–162 corresponding to anhydro-5,6,11-trideoxyTTX and 4,9-anhydro-8-*epi*-5,6,11-trideoxyTTX were detected in MRM mode with a collision energy set at 43 eV. 

### 3.3. NMR Spectroscopy and HR-FAB-MS

NMR spectra were obtained on an Agilent 600 MHz NMR spectrometer (Agilent Technologies, Santa Clara, CA, USA) in 0.4 mL of CD_3_COOD-D_2_O (4:96, v/v) at 20 °C. The signals of CHD_2_COOD at 2.06 ppm in the ^1^H NMR spectra and that of ^13^CD_3_COOD at 22.4 ppm in the ^13^C NMR spectra were used as the internal references. Signals were assigned based on the analyses of the COSY, TOCSY (mixing time 80 ms), HSQC, HMBC, and NOESY1D spectra. HR-FAB-MS (positive, matrix:glycerol) were recorded by a JEOL JMS700 MS Station (JEOL, Akishima, Japan). 

### 3.4. Quantitative/Qualitative Analysis of TTX Analogs in the Newt and Puffer Fish

Sample solutions were prepared from the pooled whole body of *Cynops ensicauda popei *and the ovary of the puffer fish, *Fugu poecilonotus*, which were captured in Yamaguchi prefecture, Japan. The toxins were extracted from homogenized tissues with two portions of 0.2 M acetic acid (v/v), heated for 10 min in boiling water. The extract was centrifuged for 15 min at 15,000 rpm at 4 °C, and the supernatant was then diluted with five volumes of water and then defatted with hexane. The extract, adjusted to pH 6.0 by 1 M NaOH, was loaded on an activated charcoal column equilibrated with water. After the column was washed with water, TTXs were eluted with acetic acid/EtOH/water (2:50:49, v/v). Volatiles were removed using a rotary evaporator, and the resulting residue was dissolved in 0.05 M acetic acid. An aliquot of this solution was subjected to LC/MS in SIM and MRM modes. 5,6,11-trideoxy-type analogs were quantified in MRM mode using the standard curve drawn for 5,6,11-trideoxyTTX to avoid overlapping with some of the other compounds. Other analogs were quantified in SIM mode using the standard curves drawn for TTX. 4,9-Anhydro-5,6,11-trideoxyTTX is not clearly distinguished from 4,4a-anhydro-5,6,11-trideoxyTTX by LC/MS. Thus, these analogs were quantified together as anhydro-5,6,11-trideoxyTTX. 5,6,11-TrideoxyTTX and 8-*epi*-5,6,11-trideoxyTTX were not clearly distinguished from each other by HILIC/MS because they have almost the same retention times and fragmentation patterns. ^1^H NMR was used to identify these analogs in the corresponding fractions.

## 4. Conclusions

In this study, four new TTX analogs, 8-*epi*-5,6,11-trideoxyTTX, 4,9-anhydro-8-*epi*-5,6,11-trideoxyTTX, 1-hydroxy-8-*epi*-5,6,11-trideoxyTTX, and 1-hydroxy-4,4a-anhydro-8-*epi*-5,6,11-trideoxyTTX, were isolated from the newt *Cynops ensicauda popei*, and their structures were determined. They are the first 8-*epi*-type analogs of TTX from a natural source and they seem to be specific to newts. In this study, we found considerable differences in the analog profiles of the newt and the puffer fish that may indicate differences in the biosynthesis, metabolism, or accumulation systems of TTXs in these two species. We will continue screening for new TTX-related compounds to reveal the biosynthetic pathway of TTX in marine and terrestrial organisms. 
